# Population genomics of *Drosophila suzukii* reveal longitudinal population structure and signals of migrations in and out of the continental United States

**DOI:** 10.1093/g3journal/jkab343

**Published:** 2021-10-02

**Authors:** Kyle M Lewald, Antoine Abrieux, Derek A Wilson, Yoosook Lee, William R Conner, Felipe Andreazza, Elizabeth H Beers, Hannah J Burrack, Kent M Daane, Lauren Diepenbrock, Francis A Drummond, Philip D Fanning, Michael T Gaffney, Stephen P Hesler, Claudio Ioriatti, Rufus Isaacs, Brian A Little, Gregory M Loeb, Betsey Miller, Dori E Nava, Dalila Rendon, Ashfaq A Sial, Cherre S Bezerra da Silva, Dara G Stockton, Steven Van Timmeren, Anna Wallingford, Vaughn M Walton, Xingeng Wang, Bo Zhao, Frank G Zalom, Joanna C Chiu

**Affiliations:** 1 Department of Entomology and Nematology, College of Agricultural and Environmental Sciences, University of California, Davis, Davis, CA 95616, USA; 2 Florida Medical Entomology Laboratory, University of Florida Institute of Food and Agricultural Sciences, Vero Beach, FL 32603, USA; 3 Laboratory of Entomology, Embrapa Clima Temperado, BR 392 Km 78, Caixa Postal 403, Pelotas, RS 96010-971, Brazil; 4 Tree Fruit Research and Extension Center, Washington State University, Wenatchee, WA 99164, USA; 5 Department of Entomology and Plant Pathology, North Carolina State University, Raleigh, NC 27601, USA; 6 Department of Environmental Science, Policy and Management, University of California, Berkeley, CA 94720, USA; 7 UF IFAS Citrus Research and Education Center, University of Florida, Lake Alfred, FL 32603, USA; 8 School of Biology and Ecology, University of Maine, Orono, ME 04469, USA; 9 Horticultural Development Department, Teagasc, Ashtown, Dublin 15, Ireland; 10 Department of Entomology, Cornell AgriTech, Cornell University, Geneva, NY 14850, USA; 11 Technology Transfer Centre, Fondazione Edmund Mach, Via E. Mach, 1, 38010 San Michele all’Adige (TN), Italy; 12 Department of Entomology, Michigan State University, East Lansing, MI 48824, USA; 13 Department of Entomology, University of Georgia, Athens, GA 30602, USA; 14 Department of Horticulture, Oregon State University, Corvallis, OR 97331, USA; 15 United States Department of Agriculture Agricultural Research Service, Beneficial Insects Introduction Research Unit, Newark, DE 19713, USA

**Keywords:** *Drosophila suzukii*, spotted-wing drosophila, invasion genomics, population structure, genetic diversity

## Abstract

*Drosophila suzukii*, or spotted-wing drosophila, is now an established pest in many parts of the world, causing significant damage to numerous fruit crop industries. Native to East Asia, *D. suzukii* infestations started in the United States a decade ago, occupying a wide range of climates. To better understand invasion ecology of this pest, knowledge of past migration events, population structure, and genetic diversity is needed. In this study, we sequenced whole genomes of 237 individual flies collected across the continental United States, as well as several sites in Europe, Brazil, and Asia, to identify and analyze hundreds of thousands of genetic markers. We observed strong population structure between Western and Eastern US populations, but no evidence of any population structure between different latitudes within the continental United States, suggesting that there are no broad-scale adaptations occurring in response to differences in winter climates. We detect admixture from Hawaii to the Western United States and from the Eastern United States to Europe, in agreement with previously identified introduction routes inferred from microsatellite analysis. We also detect potential signals of admixture from the Western United States back to Asia, which could have important implications for shipping and quarantine policies for exported agriculture. We anticipate this large genomic dataset will spur future research into the genomic adaptations underlying *D. suzukii* pest activity and development of novel control methods for this agricultural pest.

## Introduction

Over the past decade, *Drosophila suzukii* (Matsumura), also known as the spotted-wing drosophila or the Asian vinegar fly, has become an incredibly invasive pest species and a threat to soft-skinned fruit agricultural production worldwide (dos Santos *et al.* 2017). Unlike the large majority of Drosophilidae (Diptera), which preferentially breed in decaying plant material, female *D. suzukii* possess a serrated ovipositor, enabling them to lay eggs in fresh ripening soft-skinned fruits (Walsh *et al.* 2011; Walton *et al.* 2016). First described in Japan as an agricultural pest of cherries, *D. suzukii* was primarily distributed across East Asia until researchers found wild specimens in Hawaii in 1980 (Peng 1937; Kanzawa 1939; Kaneshiro 1983). In 2008, *D. suzukii* was detected in California, and by 2009 was widespread across the Western US coast (Hauser *et al.* 2009; Bolda *et al.* 2010). In the Eastern United States, *D. suzukii* first appeared in Florida in 2009 (Steck *et al.* 2009), before again rapidly spreading across the entire east coast within a few years. Meanwhile in Europe, *D. suzukii* was first detected in Spain and Italy in 2008 and rapidly spread across Europe, appearing in France, Switzerland, Austria, Germany, and Belgium by 2012. Subsequently, *D. suzukii* arrived in South America when it was detected in Brazil in 2013 (Deprá *et al.* 2014), Argentina in 2014 (Cichon *et al.* 2015), and Chile in 2015 (Medina-Muñoz *et al.* 2015). Its rapid spread across continents suggests that human transportation is likely a major factor, as eggs laid in fresh fruit are difficult to detect before shipment. Once established in a new continent, *D. suzukii* rapidly disperse to neighboring regions, aided by its ability to adapt to a wide range of climates through phenotypic plasticity (Shearer *et al.* 2016). In the Western US coastal states alone, estimated economic losses were as high as 511 million dollars per year, assuming a 20% average yield loss (Bolda *et al.* 2010). Thus, there is much interest in understanding the patterns of migration and origin of these invasive populations, as these data can be used to inform shipping and quarantine policies and to identify routes of entry.

Previous research on the population genomics of *D. suzukii* was performed using a relatively small number of molecular markers. Adrion *et al*. (2014) used six X-linked gene fragments from flies collected across the world, and detected signals of differentiation between European, Asian, and US populations. However, they found no evidence of differentiation within the 12 US populations sampled, possibly due to the limited power provided from a small number of markers. A follow-up study using 25 microsatellite loci of samples collected between 2013 and 2015 greatly improved estimations of migration patterns worldwide; the authors found evidence for multiple invasion events from Asia into Europe and the United States as well as an East–West differentiation in the seven populations sampled in the continental United States (Fraimout *et al.* 2017). However, using microsatellites alone may miss more subtle signals of population structure compared with genome-wide datasets, as increasing the number of independent loci genotyped increases accuracy of population parameter estimates, even when the number of biological samples is low (Trask *et al.* 2011; Willing *et al.* 2012; Rašić *et al.* 2014). With the advent of affordable whole-genome sequencing (WGS), it has become feasible to sequence hundreds of individuals to study population genomics, enabling improved inference of population structure using hundreds of thousands to millions of single nucleotide polymorphism (SNP) markers (Soria-Carrasco *et al.* 2014; Wu *et al.* 2019; Lee *et al.* 2019). A study of *D. suzukii* in Hawaii used double digest restriction-site-associated DNA sequencing to identify several thousand SNPs and observed population structure between islands (Koch *et al.* 2020). However, a comprehensive survey of *D. suzukii* in the continental United States using a large number of SNPs enabled by WGS has not been conducted.

In this study, we leverage the power of WGS to individually sequence hundreds of *D. suzukii* samples to determine whether U.S. populations are stratified along a north-south cline corresponding to varying winter climates, as well as to detect whether migration is freely occurring between the Eastern and Western United States. In addition, we include several populations from Asia, Europe, and Brazil to determine frequency and source of international migrations and compare genetic diversity between invasive and native populations. We expect these analyses and the large sequencing dataset will be of value in developing policies and furthering research into mitigating the harmful effects of *D. suzukii* worldwide.

## Materials and methods

### Sample collection and genomic DNA extraction

We received either flash-frozen or ethanol-preserved samples of *D. suzukii* for genomic analysis. Japanese samples were obtained from the Ehime Japanese Stock Center (strain #E-15003; MTY3; originally collected in Ehime, Japan). Hawaiian samples were taken from a small laboratory population maintained in vials that was established in 2009 from wild-caught samples in Oahu, Hawaii. All other samples were field-collected. Ethanol-preserved samples were re-hydrated in 100-μL water prior to DNA extraction. Flies were individually disrupted using a 3-mm diameter steel bead in a TissueLyser (Qiagen, Germantown, MD) for 30 s at 30 Hz in 100 μL of 2 mg/mL Proteinase K in PK buffer (MagMAX™, Thermofisher Scientific, Pleasanton, CA) before being spun down in a centrifuge for 1 min at 10,000 rpm and incubated for 2 h at 56°C. 100 μL of MagMAX DNA lysis buffer was added to each sample, followed by a 10-min incubation, before proceeding to DNA purification using a BioSprint DNA Blood Kit on a BioSprint 96 Workstation (Qiagen), using protocol “BS96 DNA Tissue” as per manufacturer’s instructions. Supplementary Table S1 contains all sample names, collection locations, and time of collection.

### Library preparation and sequencing

Illumina WGS libraries were prepared with either the Kappa HyperPlus Kit (Roche, South San Francisco, CA) (lanes 2-4) or Qiaseq FX DNA Library Kit (Qiagen) (lanes 5–8) using 50 ng of input DNA following the manufacturer’s instructions with few exceptions. With the Kappa HyperPlus Kit, DNA was fragmented at 30°C for 20 min and incubated with adapters for 1 h. A 0.6× and 0.7× size selection with AmPure XP beads (Beckman Coulter Life Sciences, Indianapolis, IN) was added after five cycles of PCR amplification with an Eppendorf Master Cycler Pro (ThermoFisher Scientific). With the Qiagen FX kit, DNA was fragmented at 30°C for 15 min and amplified with seven cycles of PCR. In both cases, DNA library concentration and fragment size were quantified on a Qubit 2.0 fluorometer with the Qubit dsDNA HS assay kit (ThermoFisher Scientific) and a Bioanalyzer High-Sensitivity DNA chip (Agilent, Santa Clara, CA). Paired-end 150 base-pair sequencing was performed by Novogene, Inc. (Sacramento, CA) on the Illumina HiSeq 4000 platform.

### Genome alignment

Raw Illumina reads were inspected for quality using fastqc version 0.11.5 (Babraham Institute, Cambridge, UK), and trimmed for low-quality bases and adapter sequences using Trimmomatic version 0.35 (Bolger *et al.* 2014), using the following parameters: Leading qscore threshold = 10, trail score threshold = 10, minimum read length = 36, and illuminaclip = 2:30:10. Reads were then aligned to the *D. suzukii* reference genome obtained from Dr. Benjamin Prud'homme (now available at GenBank accession GCA_013340165.1) (Paris *et al.* 2020) using bwa-mem version 0.7.9a (Li 2013), sorted by samtools-sort version 1.3.1 (Wellcome Trust Sanger Institute, London, UK), de-duplicated with picardtools-MarkDuplicates version 2.7.1 (Broad Institute, Cambridge, MA), and indexed with samtools-index version 1.3.1. Samtools-stats was used to obtain summary statistics of BAM files. Based on consistently low mapping rates for all samples (<70%), the Dandong, China population and one of two Watsonville, California, US collections were excluded from analysis.

### COX2 sequence analysis to confirm species identification

The *D. suzukii* mitogenome sequence and ten *D. pulchrella* COX2 sequences were downloaded from NCBI. The *Drosophila subpulchrella* mitogenome was identified by running BLAST with the *D. suzukii* mitogenome against the *D. subpulchrella* genome assembly (GCA_014743375.2), and annotated using MITOS2 (Bernt *et al.* 2013). COX2 sequences from all our *D. suzukii* samples were identified by aligning raw reads to the *D. suzukii* mitogenome (GenBank accession KU588141.1), filtering out any read pairs where one of the reads was unmapped (samtools view –f 2 –F 4). Variants were called with Freebayes version 1.1.0 in haploid mode (Garrison and Marth 2012), and fasta sequences were extracted with bcftools-consensus version 1.10.2 (Wellcome Trust Sanger Institute). Publicly available COX2 sequences of *D. pulchrella, D. suzukii, D. biarmipes, D. lutescens, D. mimetica, and D. melanogaster* were downloaded from GenBank (Supplementary Table S2).

All COX2 sequences were aligned with the ClustalOmega web portal (Madeira *et al.* 2019) resulting in a 720 base pair alignment. Forty-seven haplotypes were identified using DNASP version 6.12.03 (Rozas *et al.* 2017). MEGA version 10.1.8 was used to identify the best nucleotide substitution model based on the Bayesian Inference Criterion score (Kumar *et al.* 2018). While the best scoring model was the Tamura 3-parameter model (T92) + invariant sites (+I) + gamma distributed rates (+G), we decided to use the second best scoring model T92+G, as combining +I and +G may be problematic due to correlated parameters (Jia *et al.* 2014). Using MEGA, initial trees for the heuristic search were obtained automatically by applying Neighbor-Joining and BioNJ algorithms to a matrix of pairwise distances estimated using the Tamura 3 parameter model and then selecting the topology with superior log likelihood value. Bootstrap percentages were generated from 500 replicate runs. Five categories were used in the discrete Gamma distribution to model evolutionary rate differences among sites (+*G*, parameter = 0.1066).

### Principal component and admixture proportions analysis

Genotype likelihoods (GLs) were estimated from aligned reads with ANGSD version 0.935 (Korneliussen *et al.* 2014) using the samtools model with the following parameters: minimum mapping quality = 20, minimum base quality = 20, uniquely mapping reads only, excessive mismatch adjustment coefficient = 50. Using a *P*-value cutoff of 1E-6 and a minimum minor allele frequency = 0.05, ANGSD identified 4,955,596 SNPs. Linkage disequilibrium was estimated from the GLs and SNP list using ngsLD version 1.1.1 (Fox *et al.* 2019) to a maximum SNP pairwise distance of 100 kb, and a 1% subsample was used to estimate and plot LD decay using the accompanying Rscript “fit_LDdecay.R” with the following parameters: bin size = 100 bp, bootstraps = 100, fit level = 10, and a recombination rate = 2.3cM, based on the average rate in *D. melanogaster* (Fiston-Lavier *et al.* 2010; Comeron *et al.* 2012). Based on these plots (Supplementary Figure S1), GLs were pruned to one SNP per 1 kb or 5 kb, leaving 209,243 and 49,127 SNPs, respectively.

Principal component analysis was performed using PCAngsd version 1.0 (Meisner and Albrechtsen 2018). PCAngsd also reports the optimal number of clusters that describes the population structure (k), based on a minimum average partial (MAP) test. For each region, PCangsd was run five times with a random starting seed value and the soft upper search bound of alpha = 500; the run with the highest log likelihood was kept. Admixture proportions were then estimated using NGSadmix version 32 (Skotte *et al.* 2013). For each sample set, the run with the highest log likelihood from five independent runs was kept. Analysis of each region used k based on the number of optimal PCs + 1 reported from PCangsd. Analysis of all samples combined as well as four subsampled datasets was performed with k ranging from 3 to 10. As there was no notable difference in results using the 1 or 5 kb pruned dataset, only the 1 kb pruned results are reported here.

### Treemix admixture graphs and F3/F4 statistics

Based on admixture proportion estimates and PCA, samples were grouped into the following clusters: Eastern United States, Western United States, Hawaii, Brazil, Ireland, Italy, South Korea, and Japan. One Eastern US population from Alma Research Farm, Georgia (AR) was excluded as it unexpectedly clustered with the Western US populations. To root the population trees, two sister species of *D. suzukii* were downloaded and aligned to the *D. suzukii* genome; *D. biarmipes* (SRA accession SRX097584) and *D. subpulchrella* (SRA accession SRX8970519). GLs and SNPs were called with ANGSD as described above and pruned by only keeping SNPs found originally in the 1-kb pruned dataset used for PCA and admixture analysis. As X-linked and autosomal SNPs may have different phylogenetic signals, X-linked SNPs were excluded. As Treemix requires genotypes to be called, PCAngsd was used to call genotypes from the GLs with a 95% posterior probability cutoff using estimated inbreeding coefficients as a prior. When looking at the distribution of fraction of missing genotypes per site, we observed a peak at 10%, and decided to exclude any sites with greater than 20% missing data across samples, consistent with cutoffs in other studies (Brandenburg *et al.* 2017; Zecca *et al.* 2019). We also excluded sites for which data are completely missing within any one cluster as required by Treemix, leaving 29,145 SNPs for analysis.

Treemix version 1.13 (Pickrell and Pritchard 2012) was used to generate population admixture graphs with inferred migrations. Between 0 and 10 migrations were tested, each with 100 bootstraps calculated using a resampling block size of 500 SNPs, with global tree rearrangements and standard error estimation of migration weights enabled. The bootstrap run with maximum likelihood for each migration tested was used for plotting. To estimate support for migration edges, Treemix was also used to calculate F3 and F4 statistics using a resampling block size of 500 SNPs to estimate standard error and *Z*-scores. The F3 statistic tests if population A’s allele frequencies are a result of mixture of allele frequencies from populations B and C. A significantly negative value of F3(A; B, C) supports admixture of B or C into A. The F4 statistic measures correlations in allele frequencies between populations A and B versus populations C and D. F4(A, B; C, D) is expected to be zero under no admixture. Assuming the tree ((A, B),(C, D)) exists, a significantly positive value suggests gene flow between A and C or B and D, while a significantly negative value suggests gene flow between B and C or A and D. By setting one of these populations to be an outgroup where no admixture is expected, it is possible to infer which population pair experienced admixture. We used a *Z*-score cutoff of 2 or −2 to determine if a value was significantly positive or negative.

### Estimation of F_ST_, nucleotide diversity, and Tajima’s D

Site allele frequencies were estimated for each cluster from the largest 20 contigs of the genome assembly with ANGSD version 0.933 using the samtools GL model, filtering for a minimum mapping quality = 20 and minimum base quality = 20. The subprogram real SFS from ANGSD was used to estimate a two-dimensional folded SFS for all pairs of clusters and to calculate the global weighted Fst for each cluster pair. RealSFS was also used to estimate one-dimensional folded SFS for each cluster in order to calculate nucleotide diversity and Tajima’s D per site. The command ANGSD/thetaStat do_stat was used to bin these summary statistics in 20-kb windows across the genome.

## Results

### Population structure exists between continents as well as within the United States and Europe

**Figure 1:**
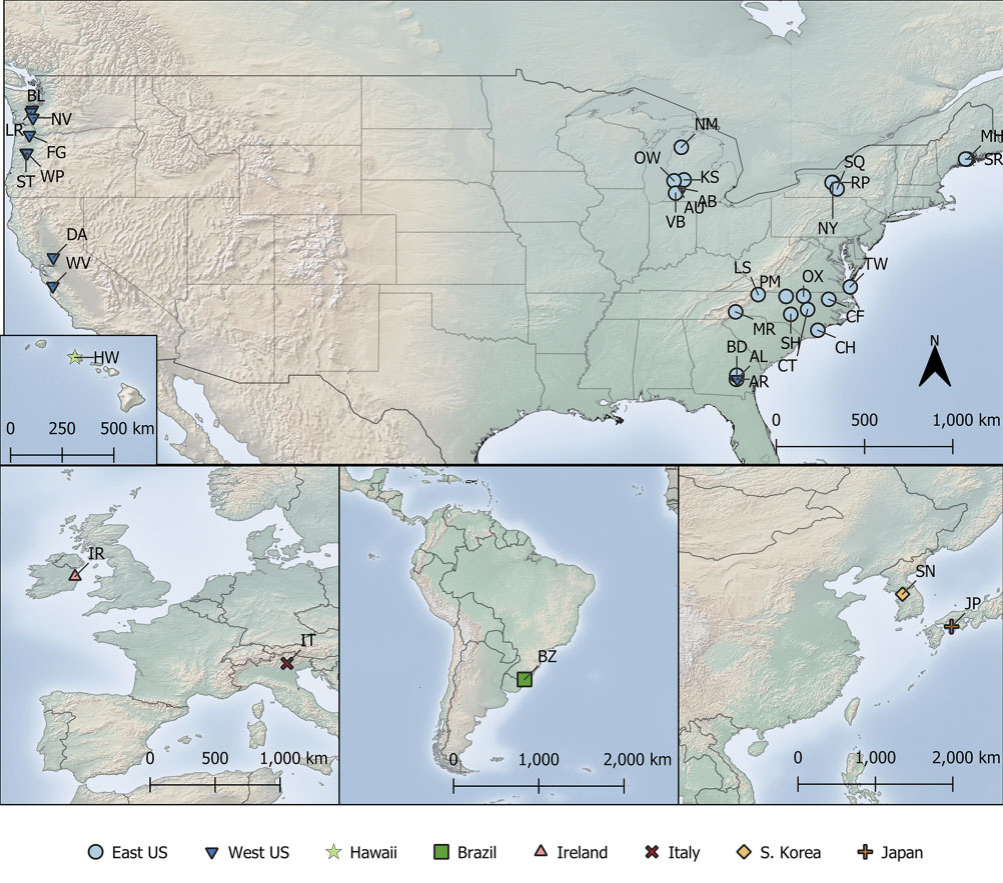
Sampling sites of *Drosophila suzukii* populations. Sampling sites from the United States, Europe, Brazil, and Asia. Labels indicate population code; colors and symbols depict population clusters as determined using PCangsd and NGSadmix. Note site AR has been labeled as “West US” based on clustering results. Between 5 and 10 flies per site were collected for WGS. Refer to Supplementary Table S1 for details of collection sites.

To determine if population structure exists in *D. suzukii* living in recently invaded locations, we sequenced wild-caught individual *D. suzukii* flies collected from the continental United States, Brazil, Ireland, Italy, South Korea, and China, as well as a laboratory strain from Hawaii and Japan (Figure 1, Supplementary Table S1). After aligning sequences to the reference genome, we found that average read coverage was low for some individuals and populations, with mean coverage per cluster ranging from 5-11X (Supplementary Table S1). As low coverage can cause biases in genotype calling, we used methods that implemented genotype likelihoods wherever possible.

We first used PCA and admixture proportion estimates to search for signs of population structure. When examining our Asian samples, we were surprised to discover that all the Namwon, South Korea samples as well as one Sancheong, South Korea sample clustered tightly with the Kunming, China population, rather than with the rest of the Sancheong samples (Supplementary Figures S2C and S3D). As several sister species to *D. suzukii* with similar morphological appearances occupy the same geographic ranges (Takamori *et al.* 2006), we performed a phylogenetic analysis using the mitochondrial COX2 gene sequence to evaluate species identity (Supplementary Figure S4). Based on phylogenetic inference, we determined that the Namwon, South Korea samples; Kunming, China samples; and one Sancheong, South Korea sample may actually be *D. pulchrella*. For this reason, these samples were excluded from further analyses.

As sampling was heavily concentrated in the United States, we first conducted PCA and admixture proportion estimation on each broad geographical region separately before analyzing all populations together (Supplementary Figures S2 and S3). Among the Eastern US samples, PCA did not separate samples by state or latitude, and no distinct populations emerged in admixture plotting at multiple clustering values (k). Among the Western US samples, both the first principal component and varying values of k for admixture proportions separates Hawaii from the other sample sites; however, higher values of k and principal components do not further partition the remaining Western US samples. Thus, it appears there is likely no strong population structure in a north to south cline in the United States. Using a similar approach, we see that in the European samples, collections from Ireland and Italy partition as separate clusters in the first PC and when *k* = 2 in admixture plotting. We also observe that samples from Asia partition into Japan and South Korea, which is unsurprising as the Japanese samples originate from a laboratory population.

**Figure 2:**
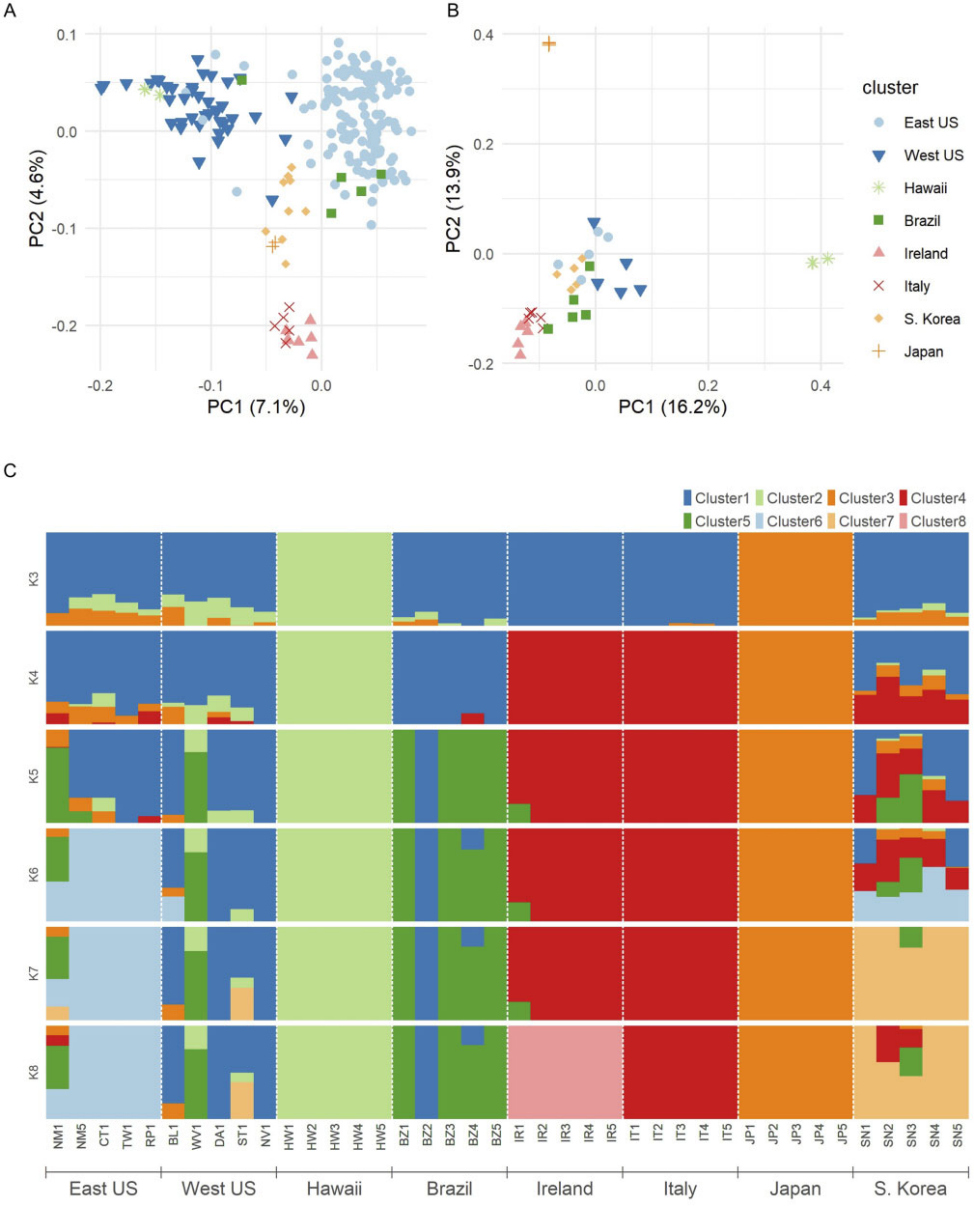
Population structure of *Drosophila suzukii* populations. (A) First two principal components plotted of all samples based on 154,271 SNPs. Note several “Eastern United States” samples representing Alma Research Farm, Georgia, as well as one Brazilian sample, clustering with the Western United States. Percent variation of the data captured by each component indicated in axis labels. (B) First two principal components of subsampled dataset, using five individuals per cluster. (C) Posterior probability of cluster identity using NGSadmix calculated from 152,876 SNPs, using between three and eight clusters. Samples labeled by name and cluster (see Supplementary Table S1).

We then used PCA to analyze all samples together to examine how differentiated invasive populations were from each other and from the ancestral Asian samples (Figure 2A). As subtler signals can be obscured by unequal population sampling (McVean 2009; Toyama *et al.* 2020), we also analyzed a reduced dataset by subsampling five individuals from each region (Figure 2, B and C). When using all samples, the first principal component separates Eastern and Western US populations, with Asian and European samples in-between. Samples from Pelotas, Rio Grande do Sul, Brazil, appear more related to Eastern US samples, although one individual clusters more with the Western US flies. We also noticed that all samples collected from the Alma Research Farm (AR), Georgia clustered with the Western rather than Eastern US samples, despite two other Georgia sites nearby that followed the expected pattern. The second principal component then separates the European samples. When the data are subsampled to five individuals per cluster, the first and second components strongly separate Hawaii and Japan, respectively (Figure 2B); this signal was likely obscured by the large number of US samples when all samples are analyzed together but is expected as these two populations were laboratory strains and have likely experienced significant genetic drift relative to wild relatives.

The observations made from PCA are largely recapitulated when using subsampled data to estimate admixture at varying levels of k (Figure 2C). At *k* = 3, we observe Japanese and Hawaiian samples form their own clusters, while all the wild collections form a third cluster. As *k* is increased up to 7, we see the appearance of Europe, Brazil, Eastern United States, and South Korea samples as their own clusters, before samples from Europe are split into Ireland and Italy at *k* = 8. We notice increased variability in cluster assignment in the US populations, particularly when subsampling, which likely reflects the large sample size and high within-population diversity (Supplementary Figure S5). However, analysis using all individuals still clearly supports Eastern and Western US samples as distinct genetic populations (Supplementary Figure S6). In addition, we also see that the AR Georgia population again clusters with the Western United States. As we were unsure if this could be the result of a very recent migration or mislabeled samples, we decided to exclude this population from further analyses.

**Figure 3:**
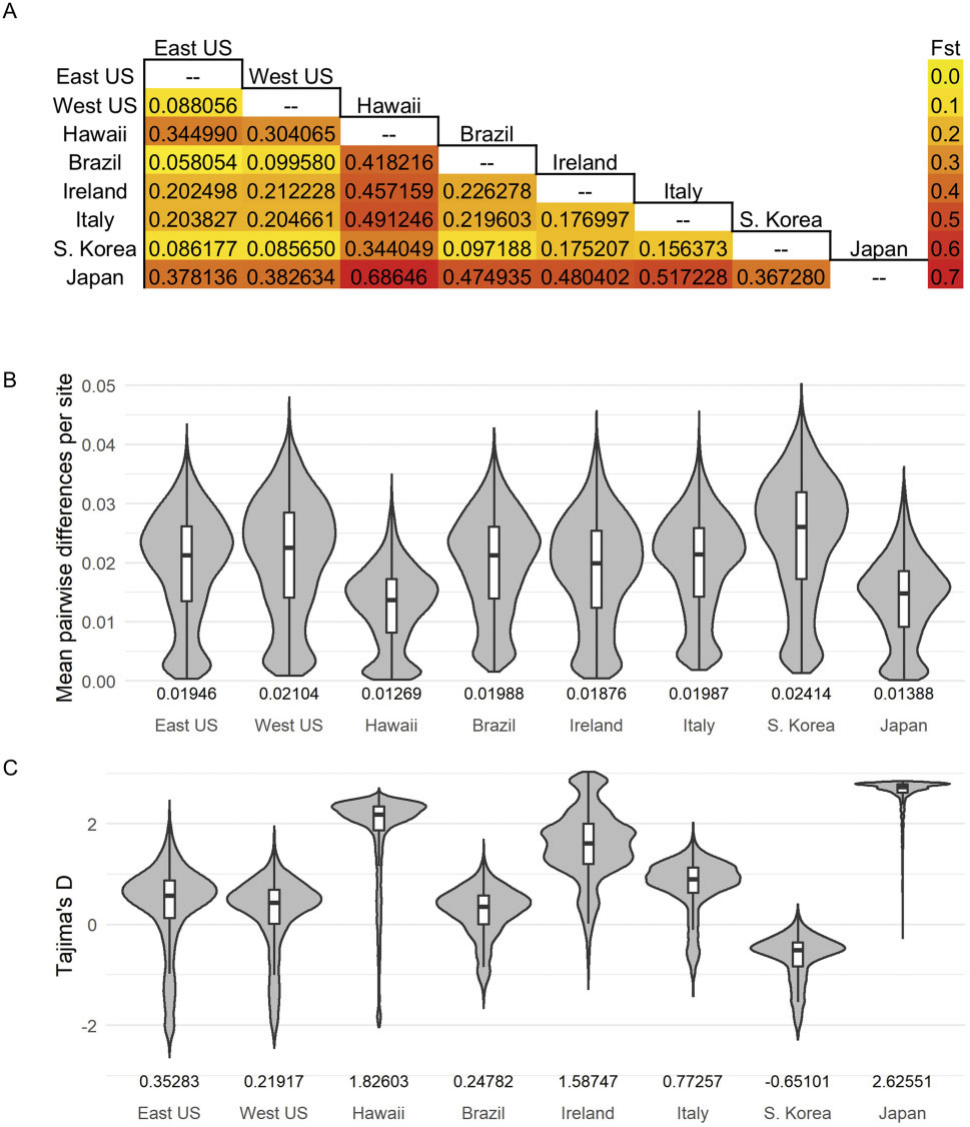
Population summary statistics of sampled *D. suzukii* populations. (A) Pairwise weighted Fst calculated from the largest 20 contigs of the reference genome between populations. (B) Pairwise nucleotide diversity distribution and (C) Tajima’s D distribution, calculated in 20 kb intervals across the largest 20 contigs of the reference genome (7237–7238 blocks). Boxplots depict median, first and third quartiles. Average values labeled along the *x*-axis.

To further quantify the amount of differentiation present between regions, we estimated Fst values between regions using the 20 largest contigs, spanning all 4 chromosomes and covering 54% (145 Mb) of the reference genome (Figure 3A). Three general levels of differentiation were apparent based on this analysis. As expected, Fst between Hawaii or Japan to any wild population was high (>0.30). Irish and Italian populations had intermediate levels of differentiation with the other wild populations and with each other (0.15–0.30), whereas Fst values between Brazil, South Korea, and both US clusters were lower (0.05–0.10). These groupings broadly match those observed from PCA (Figure 2, A and B).

### Repeat migrations to and from the United States have occurred over large geographic distances

**Figure 4:**
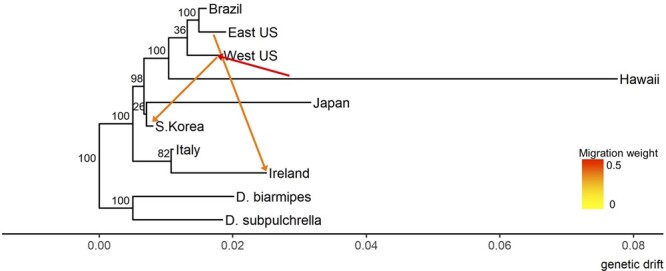
Maximum likelihood admixture graph based on allele frequencies allowing six migrations. The three strongest migrations are shown, colored by admixture proportion; Hawaii to Western United States (0.410, SE = 0.069, *P* = 1.10E-9), Eastern United States to Ireland (0.253, SE = 0.027, *P* = 0.0), and Western United States to South Korea (0.231, SE = 0.036, *P* = 5.4E = 11). Nodes labeled with jackknife bootstrap confidence percentages obtained from 100 replicates.

While PCA and admixture proportion estimates were able to identify population clusters, they are unable to provide more detailed depictions of population history or migration events. To estimate the population history of these invasive populations, we used Treemix to generate a population admixture graph with inferred migration events based on co-variance of allele frequencies between clusters, testing models allowing between 0 and 10 migrations (m) (Supplementary Figure S7). We found that the model using six migrations captured the most variance of the data (99.6%) (Figure 4). Residuals of the model at m = 6 are within ±5 standard errors between populations, suggesting the model fits the data well, despite the variance of Hawaii with itself appearing less well modeled (Supplementary Figure S8). The strongest signal of admixture was found in the Western United States, with an estimated Hawaiian admixture proportion of 41.0% (SE = 6.9%, *P* < 0.05), and was also observed in most models (m = 4–8,10) (Supplementary Table S5). To formally test for admixture, we used the F3 admixture statistic in the form F3(Western United States; Hawaii, popX) where popX represents any third population, and found significantly negative values for all populations (*Z*-score < −2), strongly supporting admixture of Hawaii into the Western United States (Supplementary Table S3). We also used the F4 statistic, using the form F4(A, B; C, outgroup) such that a negative value supports “B” and “C” admixture, whereas a positive value supports “A” and “C” admixture, assuming no migration occurred between the outgroup and either A or B. Using either *D. biarmipes* or *D. subpulchrella* as the outgroup, the tests F4(Western United States, Brazil; Hawaii, outgroup) and F4(Western United States, Eastern United States; Hawaii, outgroup) were significantly positive (Z score > 2), again supporting this admixture (Supplementary Table S4). Thus, the Western US population sampled is composed of nearly equal ancestry from a Hawaiian ancestor and the common ancestor of the US/Brazil populations. As Treemix assigns the edge with smaller weight to be the “migrant” edge by default, it may be unidentifiable whether the Hawaiian ancestor or the US/Brazil common ancestor should be called the migration source.

We also observed two countries with US admixture in the m = 6 model. Ireland had an Eastern US admixture of 25.3% (SE = 2.7%, *P *< 0.05), although at varying values of “m” the source of this admixture fluctuates between the Eastern United States, Brazil, or the Eastern US/Brazil ancestor. However, in all cases, the admixture strength and significance remain consistent (Supplementary Table S5). While no F3 statistic support was found, the F4 statistics (Western United States, Brazil; Ireland, outgroup) and (Western United States, Eastern United States; Ireland, outgroup) were significantly negative, supporting Ireland’s Eastern US/Brazilian and European ancestry. As the US/Brazilian admixture weight is much less than the European admixture weight, this was likely due to a migration event from the Americas into Irish populations.

The other out-of-US admixture event, from the Western United States to South Korea (admixture 23.1%, SE = 3.6%, *P* < 0.05), was seen when m = 6, 8, and 10. F3 statistics (South Korea; Western United States, Italy/Ireland/Japan) all have significantly negative values, and the F4 statistics (Western United States, Eastern United States; South Korea, outgroup) and (Western United States, Brazil; South Korea, outgroup) are significantly positive, supporting a Western US/South Korea admixture. However, using nine migration edges Treemix reported the reverse direction; as F3 and F4 statistics cannot easily infer directionality, more heavily sampling of the Asian populations or alternate methods may be needed to determine whether flow is occurring in both directions.

### Invasive populations have experienced little loss in genetic diversity

To determine if invasive populations have experienced loss in genetic diversity, we used the software ANGSD to estimate average pairwise nucleotide diversity in 20 kb increments across the 20 largest contigs of genome for each population. Invasive populations can sometimes exhibit reduced levels of diversity early on in their history due to a founder effect (Nei *et al.* 1975), whereas ancestral populations tend to have the greatest amount of diversity as they have had many generations to accumulate mutations. A Welch one-way test (*F* = 1590.9, *P* < 0.05) found a significant difference in mean pairwise nucleotide diversity between clusters. We then used pairwise Games-Howell tests and found each cluster to be significantly different (*P* < 0.05), except for the Eastern United States, Brazil, and Italy when compared with each other. As Asia is the ancestral home of *D. suzukii*, it is no surprise that South Korean wild populations exhibit the highest diversity levels (Figure 3B). Similarly, the laboratory populations from Japan and Hawaii have half as much pairwise diversity as the wild South Korean population, consistent with a small laboratory population size. The invasive populations display an intermediate level between these extremes.

To assess whether invasive populations may have experienced a bottleneck or population shrinkage, we also estimated Tajima’s D in the same genomic intervals. Extremely positive values (>2) can indicate a loss of rare alleles, which can occur during a population shrinkage, whereas extremely negative values (<−2) can indicate a recent bottleneck followed by rapid expansion (Tajima 1989). A Welch one-way test again indicated significant differences in mean Tajima’s D between clusters (*F* = 45598, *P*-value < 0.05), and pairwise Games-Howell tests found all clusters to be statistically different (*P* < 0.05) except for Western US against Brazilian flies. Strains from Hawaii and Japan both had high genome-wide levels of Tajima’s D, indicative of a loss of rare alleles that can occur during a population shrinkage (Figure 3C). The remaining populations had neutral values of D, except for Ireland’s relatively high value. Based on this, we conclude there are no strong signals for a recent bottleneck, although the high genome-wide D value for Ireland suggests a recent population shrinkage. As our Irish samples were collected in 2016 only 1 year after its discovery in Ireland, we could be observing the founder’s effect in action (Gaffney 2017).

## Discussion

Based on population allele frequencies, we have shown that *D. suzukii* exhibit population structure based on region and invasion history. In the New World populations, we find that Eastern and Western US samples appear to be distinct populations. While this could be the result of continuous population variation from East through Central to the West coast, it is more likely the case that the two populations experience little gene flow due to strong geographical barriers such as the Sierra Nevada or Rocky Mountain ranges, and the fact that key target fruit crops such as cherries, raspberries, blueberries, and strawberries are primarily grown in states that we sampled (“Noncitrus Fruits and Nuts 2019 Summary” 2020). Any genetic exchange between these regions would likely be the result of human activity, such as could be the case with samples collected from Alma Research Farm, Bacon County, Georgia (AR) clustering with the Western US populations. As other nearby collections (AL, BD) failed to share this signal, the Alma research population could represent a recent and isolated migration event. Otherwise, we see little evidence of migration events or admixture between the Eastern and Western United States, which is somewhat surprising as the country’s supply of fresh blueberries, cherries, and caneberries are concentrated in a few states (Pacific Northwest, Michigan, Maine) and shipped across the country (“Noncitrus Fruits and Nuts 2019 Summary” 2020). However, recent changes to cultural management such as more frequent harvesting and post-harvest chilling may be responsible for disrupting the *D. suzukii* lifecycle and limiting cross-country transport (Schöneberg *et al.* 2021).

While we were able to detect population structure between eastern and western locations in the United States, we were surprised to discover a lack of structure on a finer scale, either based on latitude or simple geographical distance, given the large number of loci analyzed. In a similar study using 3484 SNPs in 246 Hawaiian *D. suzukii* samples, researchers were able to identify three distinct populations roughly seperated by islands (Koch *et al.* 2020). The fact that *D. suzukii* has been present in Hawaii since 1980, in addition to the isolation by island, are likely the strongest factors in providing enough genetic drift to create such differentiation. As the continental US *D. suzukii* have only been present since 2008, it may be too early for finer structure to have developed. Alternatively, continual dispersion and transportation of *D. suzukii* around the United States may be hindering the development of more local structure.

Several studies have reported a low probability of *D. suzukii* surviving when exposed to freezing temperatures, based on cold survival assays of wild-caught specimens (Dalton *et al.* 2011; Stephens *et al.* 2015), suggesting that flies collected in cold-winter regions such as Washington, Michigan, Maine, and New York could be annual migrants to the area from nearby warmer locations. The lack of north-south population structure supports the hypothesis that flies are regularly re-migrating into colder climates after the harsh winters have ended. Alternatively, flies could be tolerating winters by surviving inside human structures (Stockton *et al.* 2019), or by having evolved resistance to freezing temperatures (Stockton *et al.* 2020). Studies using *D. suzukii* collected from different locations have reported different levels of rapid cold-hardening response, suggesting that there could be regional selection present (Jakobs *et al.* 2015; Everman *et al.* 2018; Stockton *et al.* 2020). If populations in northern regions undergo strong seasonal fluctuations in allele frequencies, such as has been demonstrated in wild *D. melanogaster* populations collected in Pennsylvania (Bergland *et al.* 2014), by only sampling sites in the summer we may be missing signals of population differentiation between the north and south. Likely, some combination of these factors is responsible for the success of *D. suzukii* in these regions, and further studies will be needed to identify the causes. North-south clines in specific traits such as diapause and circadian rhythms have been previously identified in drosophilids and could be at play here as well (Schmidt *et al.* 2005; Tyukmaeva *et al.* 2011). Further analyses using methods such as those recently used to detect SNPs correlated with invasive success (Olazcuaga *et al.* 2020) could be applied to this dataset to find signals of selection.

Fst values between populations from the United States, Brazil, and South Korea were low and agree with previously published Fst estimates based on Pool-Seq data; Olazcuaga *et al.* 2020 observed that Fst between US, European, Asian, and Brazilian populations varied between 8.86 and 9.02%. However, we were surprised to see that our Italian and Irish samples had much higher values of Fst compared with the other populations, and even to each other. This discrepancy could be due to the small sample sizes we had from Europe; in this scenario, pooling larger number of samples can improve power to estimate Fst, and we instead rely on comparing the relative Fst values between populations for our analysis. High Fst values between our Japanese and Hawaiian populations were expected, however, as these have likely experienced strong drift during their time in captivity.

In general, we find that our treemix and migration results largely coincide with the proposed invasion pathway inferred from microsatellites (Fraimout *et al.* 2017), as well as a recent pre-print that re-analyzed invasion pathways with pooled sequencing data (Gautier *et al.* 2021). We see that European and US/Brazilian populations form two distinct clades, emphasizing these regions were invaded by two independent migrations from Asia. Hawaii is the first population to diverge in the Americas, followed by the Western United States, then the Eastern United States and Brazil. Additionally, in the Western United States, we detected a strong signal of admixture from Hawaii, which could be due to multiple or ongoing migration events. We also detected signals of admixture from the Eastern United States/Brazil to Ireland, which matches the predicted initial invasion pathway and suggests multiple migration events. Unique to our analysis, we recover support for admixture of Western US samples in Asia, suggesting that migrations could be ongoing in both directions. Invasive species transport is strongly associated with international trade of live plants and plant products (Chapman *et al.* 2017), and indeed agricultural export data supports the possibility of this migration as Japan receives almost 15% of all US blueberry exports, and Oregon recently became the first state to begin shipping blueberries to South Korea in 2012 (Evans and Ballen 2014). It should be noted that while Treemix infers direction of migration, the model can occasionally infer the incorrect direction, particularly when populations are closely related without an available outgroup (Pickrell and Pritchard 2012). More sampling of Asian populations are likely needed to confirm the direction of this admixture.

In conjunction with evidence of this widespread ongoing migration, we observed nucleotide diversity levels of all invasive populations (excluding laboratory populations) to be only moderately below that of the wild South Korean population, a trend also observed in Fraimout et al. (2017). Typically, recent invasion events are characterized by reduced diversity relative to the ancestral populations due to founder or bottleneck effects (Dlugosch and Parker 2008). However, successive invasion events can provide relief from any initial bottlenecks by providing increased genetic diversity. This has been observed to occur in multiple animal studies (Johnson and Starks 2004; Kolbe *et al.* 2004) and could lead to increased ability to adapt and evolve to new climates. Correspondingly, in our analysis, we did not find populations with broadly low values of Tajima’s D, suggesting little bottleneck effect. As measures to reduce impacts of invasive species are often hindered by repeated migrations (Garnas *et al.* 2016), it will be important to enforce that fruits being exported and imported internationally are free of live *D. suzukii* as required by the US Department of Agriculture, even though this species is already internationally established.

We anticipate that the genomic data provided here will prove useful in many fields of biology beyond the scope of this study. Knowledge of genetic variation and alternate alleles present within a species can be informative for the design of probes and micro RNAs (miRNAs), such as for the purpose of creating gene drives to control invasive species. Gene drive mechanisms to eliminate *D. suzukii* have been experimentally tested on multiple lines to ensure the miRNAs are broadly effective (Buchman *et al.* 2018), but a large dataset of wild population sequencing allow researchers to more confidently select target sites that are non-variable and thus susceptible to Cas9 targeting (Schmidt *et al.* 2020). Drury *et al.*, (2017) demonstrated that minor natural polymorphisms in target sites reduce gene drive effectiveness in flour beetles, and tools have been developed to help researchers design gRNAs accounting for population variation (Chen *et al.* 2020). Similarly, with the recent development of a CRISPR-Cas9 editing and RNAi knockdown protocols for *D. suzukii* (Murphy *et al.* 2016; Li and Scott 2016; Taning *et al.* 2016; Ahmed *et al.* 2020), prior knowledge of allelic variation will allow researchers to design targeting oligonucleotides more precisely to avoid loci with variability. Most recently, our dataset has been used to study sensory receptor evolution in *D. suzukii*, giving insights into its evolution toward becoming a major agricultural pest (Durkin *et al.* 2021). Other future uses of this trove of genomic data could involve insecticide resistance studies or the development of diagnostic assays for rapid detection in the field.

## Data availability

Raw Illumina reads have been deposited to the NCBI Sequence Read Archive (SRA) and can be found under BioProject accession number PRJNA705744. Supplementary Table S2 contains GenBank accessions of COX2 sequences used. Supplementary Tables S3 and S4 contain F3 and F4 statistics for all populations calculated by Treemix. Supplementary Table S5 contains all migrations inferred from Treemix at all migration values tested. Supplemental figures and tables have been uploaded to GSA figshare portal: https://doi.org/10.25387/g3.16655185. Scripts used to run all analyses can be found at www.github.com/ClockLabX/Dsuz-popgen.
